# Possibility of determining high quantitative fecal occult blood on stool surface using hyperspectral imaging

**DOI:** 10.1007/s00535-024-02163-2

**Published:** 2024-10-23

**Authors:** Hiroaki Ikematsu, Yohei Takara, Keiichiro Nishihara, Yuki Kano, Yuji Owaki, Ryuji Okamoto, Takahisa Fujiwara, Toshihiro Takamatsu, Masayuki Yamada, Yutaka Tomioka, Nobuyoshi Takeshita, Atsushi Inaba, Hironori Sunakawa, Keiichiro Nakajo, Tatsuro Murano, Tomohiro Kadota, Kensuke Shinmura, Yoshikatsu Koga, Tomonori Yano

**Affiliations:** 1 Division of Science and Technology for Endoscopy, Exploratory Oncology Research and Clinical Trial Center, National Cancer Center, Kashiwa, Japan; 2 Department of Gastroenterology, IMSUT Hospital, The Institute of Medical Science, The University of Tokyo, 4-6-1 Shirokanedai, Minato-ku, Tokyo, 108-8639 Japan; 3 EBA JAPAN Co., Ltd., Tokyo, Japan; 4 Department of Gastroenterology and Endoscopy, National Cancer Center Hospital East, Kashiwa, Japan; 5 Health and Medical Research Institute, National Institute of Advanced Industrial Science and Technology, Tsukuba, Ibaraki Japan; 6 Medical Device Innovation Project Management Office, National Cancer Center Hospital East, Kashiwa, Japan; 7 Division of Medical Device Innovation Support, National Cancer Center Hospital East, Kashiwa, Japan; 8 Department of Strategic Programs, Exploratory Oncology Research and Clinical Trial Center, National Cancer Center, Kashiwa, Japan

**Keywords:** Colorectal cancer, Fecal occult blood, Colorectal cancer screening, Hyperspectral imaging

## Abstract

**Background:**

Fecal immunochemical tests are commonly performed for colorectal cancer screening. Instant fecal occult blood measurement in toilet bowel movements would improve convenience. Hyperspectral imaging (HSI) enables the nondestructive evaluation of materials that are difficult to assess visually. This study aimed to determine whether HSI could be used to identify fecal occult blood on stool surfaces.

**Methods:**

The study included 100 patients who underwent colonoscopy, divided into groups A and B (50 patients, each) for creating a discriminant algorithm and validating the accuracy of the algorithm, respectively. In group A, 100 areas were randomly selected from the stool surface, and the fecal occult blood quantitative values were measured and photographed using a hyperspectral camera (cutoff: > 400 ng/mL). A discriminant algorithm image was created to extract spectral feature differences obtained from HSI via machine learning. In group B, 250 random areas were evaluated and compared to fecal occult blood quantitative values, measuring sensitivity, specificity, accuracy, positive predictive value (PPV), and negative predictive value (NPV).

**Results:**

Groups A and B comprised 28 and 26 patients with cancer, respectively. Cancer detection sensitivity at the 400 ng/mL cutoff was 67.9% and 42.3% in groups A and B, respectively. The discriminant algorithm image exhibited high accuracy in group A (sensitivity; 77.1%, specificity; 96.9%, accuracy; 90.0%, PPV; 93.1%, NPV; 88.7%). In group B, the sensitivity, specificity, accuracy, PPV, and NPV were 83.3, 92.9, 90.8, 76.3, and 95.3%, respectively.

**Conclusion:**

HSI can effectively discriminate high quantitative fecal occult blood, highlighting its potential for improved colorectal cancer screening.

## Introduction

Colorectal cancer is the third most common cancer in terms of incidence and the second most common one in terms of mortality worldwide [1]. The results of the National Polyp Study in the United States indicated that removal of adenomatous lesions contributes to reducing colorectal cancer morbidity and mortality, and the importance of colorectal cancer screening in preventing colorectal cancer is widely recognized [2].

Screening methods vary depending on the country, but among them, fecal immunochemical tests (FIT) for fecal occult blood are widely used worldwide [3–6]. FIT is superior in terms of non-invasiveness, cost-effectiveness, and ease of use and is a proven effective method for mortality reduction [7, 8]. A FIT cutoff value of 100 ng/ml has been adopted in many countries as the suitable value for screening [4, 9]. Moreover, patients with advanced colorectal cancer have been reported to show high quantitative values on FIT [10, 11]. However, one of the drawbacks of FIT is that it requires scraping a sample from the stool and storing this collected sample in a cool, dark place. This could be one of the reasons for the reduced screening rate.

In this study, we focused on hyperspectral imaging (HSI) as a potential technology for the instantaneous imaging of hemoglobin (Hb) in stool. All materials have the property to reflect or absorb certain wavelengths of light when irradiated, and analyzing those wavelengths will enable us to determine the spectrum of the object. HSI is a next-generation optical measuring instrument that performs spectroscopic analysis on every pixel of a two-dimensional image and can acquire more than 100 types of spectroscopic information on every pixel [12]. It enables the visualization of phenomena beyond human color perception and the evaluation of physical properties in a nondestructive, noncontact manner, yielding numerous research results in the field of medicine [13–15]. If the Hb in stool can be photographed using HSI, instant determination of the presence or absence of fecal occult blood during daily bowel movement in the toilet can be expected without the hassle of collecting and storing stool samples.

As a first step, we aimed to determine whether HSI could be used to identify high quantitative fecal occult blood in the stool surface.

## Methods

### Study design and approval

This was a single-center, prospective, observational study. The study protocol was approved by the Institutional Review Board of the National Cancer Center (approval number: 2020–126). Written informed consent was obtained from all the participants. The study was conducted in accordance with the World Medical Association Helsinki Declaration.

### Participants

The study included 100 patients who underwent lower gastrointestinal endoscopy with in-hospital laxatives at the National Cancer Center Hospital East between October 31, 2021, and April 30, 2022, and who provided their consent to participate. Of these patients, the first 50 were categorized into group A for creating a discriminant algorithm, and the remaining 50 were categorized into group B for validating the accuracy rate of the discriminant algorithm. The exclusion criteria were (1) patients under 20 years of age and (2) patients with a history of bowel resection, and the discontinuation criterion was (1) patients whose collected stool showed only soluble diarrhea with no solid component. Data from patients who met the discontinuation criteria were not collected, and enrollment continued until new consent was obtained from a separate patient, resulting in a total of 100 patients included in this study.

### Stool specimen analysis using HSI and quantitative measurement of fecal occult blood

**Fig. 1:**
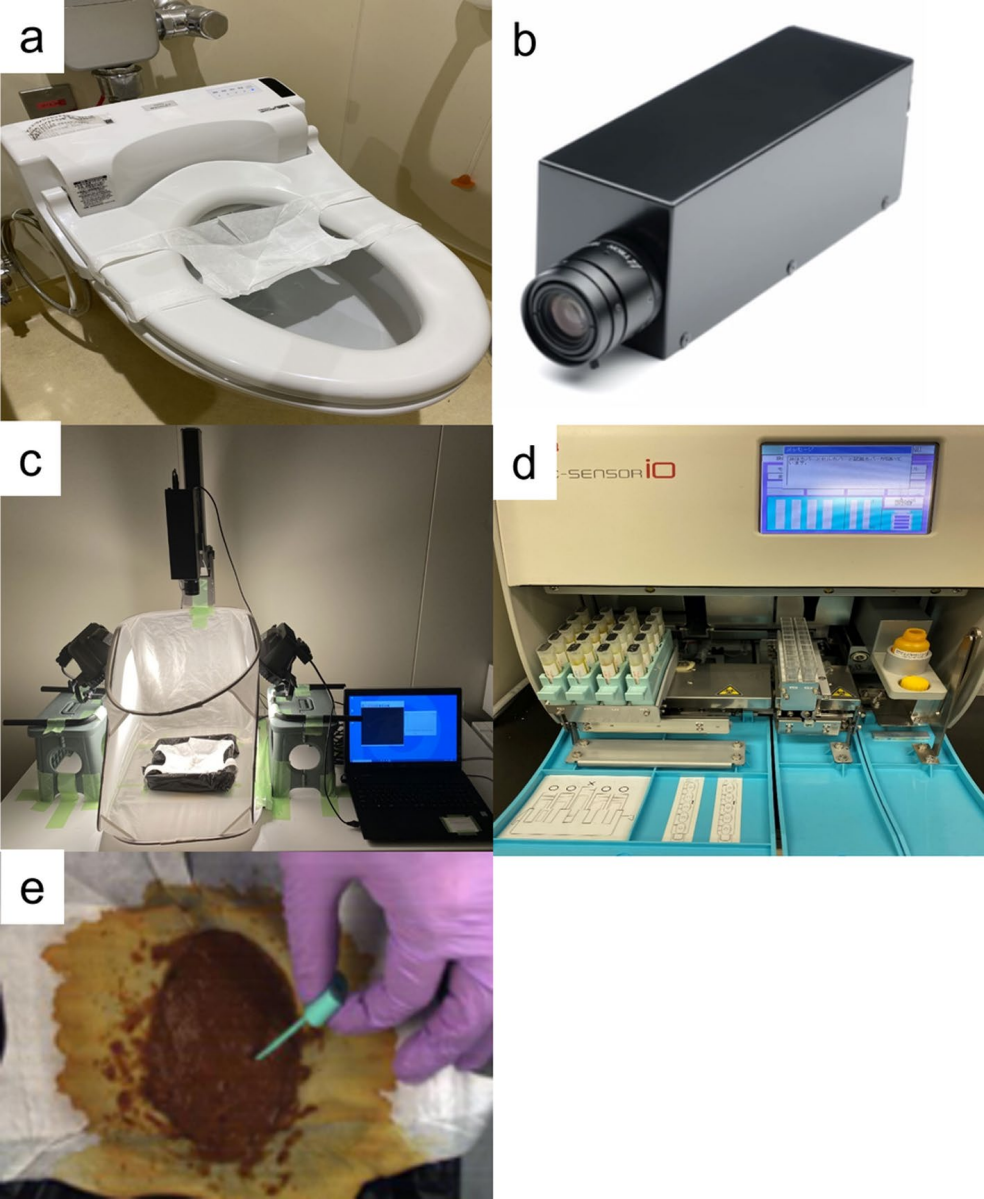
Measuring instruments and methods. **a** The Raku-Ryu Cup Wide (Takahashi Keisei Corporation), placed in a toilet bowl for stool specimen collection. **b** Hyperspectral camera (NH-7, EBA JAPAN CO., LTD.). **c** Image capture setup using NH-7. **d** OC-Auto Sampling Bottle analysis by the OC-SENSOR io (Eiken Chemical CO., LTD.). **e** Photo of fecal occult blood measurement area

The first stool specimen was collected in the hospital using a Raku-Ryu Cup Wide (Takahashi Keisei Corporation, Yamagata, Japan) stool collection cup (Fig. 1(a)). One area of the stool surface was collected by abrasion using a stool collection kit (OC-Auto Sampling Bottle; Eiken Chemical CO., LTD., Taito-ku, Tokyo, Japan) (Fig. 1e), which is used for fecal occult blood tests. Images, including the sampling area, were captured using a hyperspectral camera (NH-7; EBA JAPAN CO., LTD., Shinagawa-ku, Tokyo, Japan) with 121 continuous spectra ranging from 400 to 1000 nm at 5-nm intervals. A halogen lamp light source with a white diffuser was used to ensure consistent lighting and minimize shadows (Fig. 1b, c). Hyperspectral images were calibrated with a standard white reference to obtain reflectance data. Quantitative counts of fecal occult blood were measured using the OC-SENSOR io (Eiken Chemical CO., LTD., Taito-ku, Tokyo, Japan) (Fig. 1d).

### Construction of the discriminant algorithm

In group A, 100 areas (two areas per case) on the stool surface were randomly selected in each case, and the fecal occult blood quantitative values were measured and photographed using a hyperspectral camera. In the 50 cases (100 areas) for algorithm development, the sensitivity of lesion detection was compared at high quantification values of 400 ng/ml and 800 ng/ml, and the cutoff values for the algorithm were determined. Subsequently, fecal occult blood quantification values above the determined cutoff value were considered positive. Differences in spectral features, obtained by photographing the measurement area with a hyperspectral camera, were extracted through machine learning and other methods to create a discriminant algorithm that separates the two groups. Briefly, preprocessing steps, including smoothing, normalization, multiplicative scatter correction, and second deviation, were followed by image classification using a support vector machine with band selection methods, such as sequential backward elimination [16]. If the detected region in the analysis image was located on the stool, it was judged as positive.

### Validation of the discriminant algorithm

In group B, 5 areas were determined in each case, and a total of 250 areas were measured for fecal occult blood quantification and photographed using HSI. The 5 areas for analysis were determined by the following method: (1) cases in which the entire stool was positive on the discriminant algorithm image were defined as a whole positive pattern, and the five positive areas were selected for analysis; (2) cases in which a portion of the stool was positive were designated as partial positive pattern, and 3 positive areas and 2 negative areas were included in the analysis; (3) cases in which the entire stool was negative were designated as whole negative pattern, and 5 negative areas were included in the analysis.

Outcomes were sensitivity, specificity, positive diagnostic rate, positive predictive value (PPV), and negative predictive value (NPV) in all 250 sampling areas and in cases.

### Verification of the Hb recognition accuracy of the algorithm image

In order to verify whether the algorithm image accurately recognizes Hb instead of color recognition, and whether it can also recognize the deep part of solid stool, it was verified using the liquid and the stool model made of brown colored flour.

Petri dishes were prepared with various types of liquids (water, milk, tomato juice, tea, and coffee) and a small amount of blood was dropped into the liquid. The stool models were prepared with fresh and coagulated blood on a part of the surface and with fresh blood inside the stool model. Each was verified whether only the blood portion was imaged in the algorithm image.

### Statistical analysis

Nominal variables are expressed as frequencies and continuous variables were expressed as median with range. The Pearson X2 test or the Fisher exact test was used to analyze categoric data and compare proportions.

All *P* values were reported as two-sided, with a significance level of 0.05. All statistical analyses, including calculation of accuracy, were performed with EZR (Saitama Medical Center, Jichi Medical University, Saitama, Japan) and SAS (version 9.4), a graphical user interface for R 4.1.0 (R Foundation for Statistical Computing, Vienna, Austria). More precisely, EZR is a modified version of R commander (version 2.7–0) designed to add statistical functions frequently used in biostatistics [17].

## Results

### Baseline characteristics

The characteristics of each group of recruited patients are shown in Table 1. Sex, age, and constant use of antiplatelet or antithrombotic drugs were not significantly different between the two groups. The ratios of cancer, advanced adenoma, non-advanced adenoma, and no lesion were also not significantly different between the groups.

**Table 1:** Patient characteristics

	Group A (*n* = 50)	Group B (*n* = 50)	*p*
Sex *n* (%), male	33 (66.0)	30 (60.0)	0.679
Age, median (range)	69 (37–84)	68 (27–85)	0.446
Antiplatelet or antithrombotic drugs +	6	3	0.487
Colorectal cancer			
Any	28	26	0.841
Proximal/distal	7/21	5/21	
Advanced cancer			
Any	19	18	1.000
Proximal/distal	6/13	3/15	
Early cancer *			
Any	9	8	1.000
Proximal/distal	1/8	2/6	
Advanced adenoma **,****	4	7	0.362
Non-advanced adenoma ***, ****	10	8	0.795
No lesion	8	9	1.000

*Early cancer was defined as intramucosal cancer and submucosal invasive cancer

**Advanced adenoma: ≥ 10 mm and/or high-grade adenoma

***Non-advanced adenoma: < 10 mm and low-grade adenoma

****Advanced adenoma and non-advanced adenoma are aggregated with the highest grade of malignant lesion

### Creating the discriminant algorithm

**Fig. 2:**
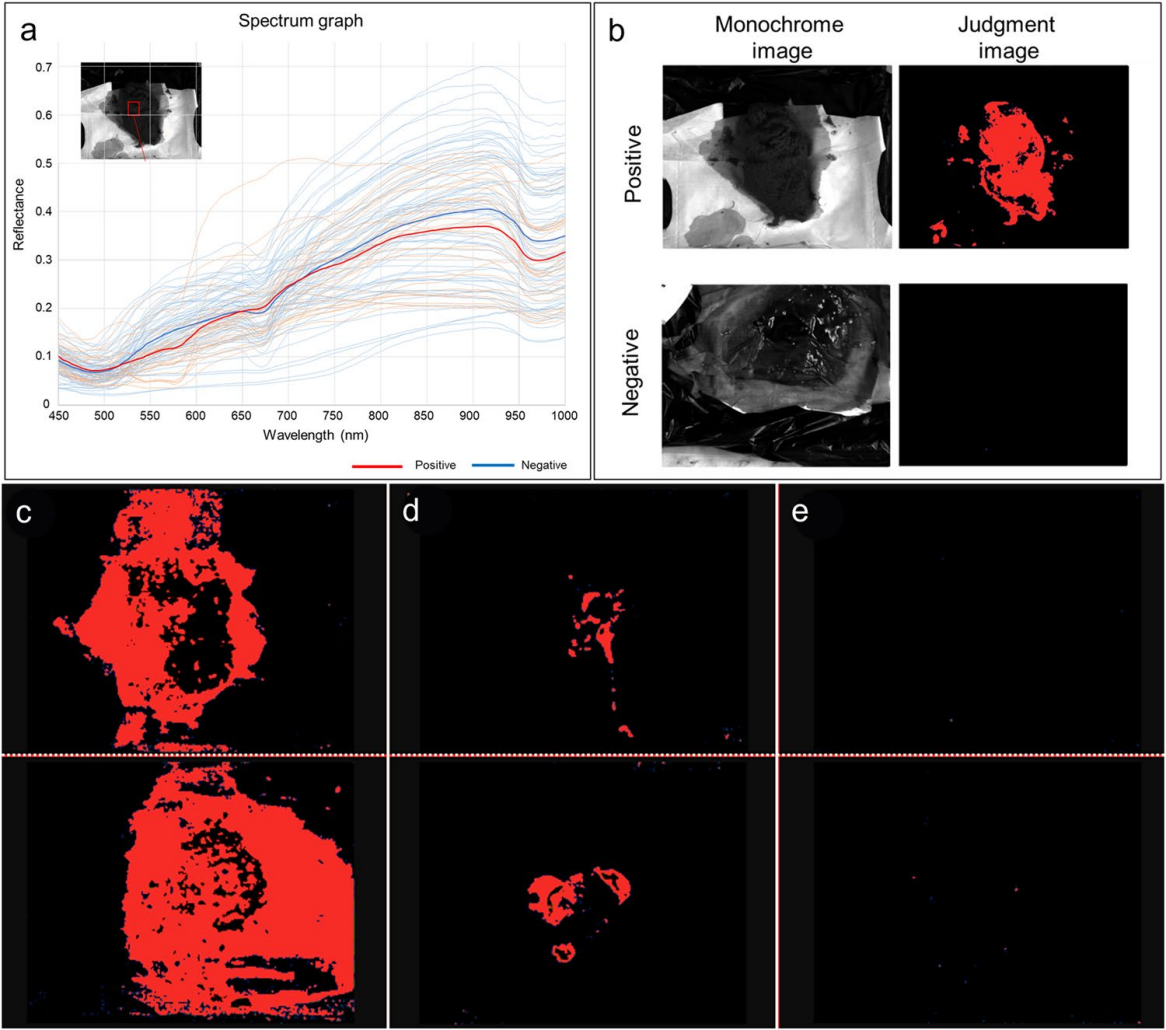
Discriminant algorithm image creation and image of validation test. **a** Reflectance spectra of each sampling area. The thick lines indicate the average of the observed spectra. **b** Representative discriminant images of positive and negative samples. **c** Whole positive pattern (cases in which the entire stool was positive on the discriminant algorithm image.) **d** Partial positive pattern (cases in which a portion of the stool was positive). **e** Whole negative pattern (cases in which the entire stool was negative)

Table 2 shows the sensitivity of each lesion according to fecal occult blood quantification at 400 ng/ml and 800 ng/ml in group A. The fecal occult blood quantification value of 400 ng/ml showed no significant difference in sensitivity to colorectal cancer or advanced adenoma compared to 800 ng/ml. Therefore, a cutoff value of 400 ng/mL was determined to minimize false negatives for cancer detection. The discriminant algorithm images were generated using machine learning to extract differences in spectral features obtained from HSI (Fig. 2a), highlighting regions with fecal occult blood quantification values > 400 ng/mL in color (Fig. 2b).

**Table 2:** The sensitivity of each lesion according to fecal occult blood quantification in group A

	Fecal immunochemical test						
	*n*	≥ 100 ng/ml		≥ 400 ng/ml		≥ 800 ng/ml	
		*n*	Sensitivity	*n*	Sensitivity	*n*	Sensitivity
Colorectal cancer							
Any	28	23	82.1	19	67.9	17	60.7
Proximal/distal	7/21	7/16	100/76.2	7/12	100/57.1	7/10	100/47.6
Advanced cancer							
Any	19	18	94.7	15	78.9	13	68.4
Proximal/distal	6/13	6/12	100/92.3	6/9	100/69.2	6/7	100/53.8
Early cancer ^*^							
Any	9	5	55.6	4	44.4	4	44.4
Proximal/distal	1/8	1/4	100/50.0	1/3	100/37.5	1/3	100/37.5
Advanced adenoma ^**, ****^	4	1	25.0	1	25.0	1	25.0
Non-advanced adenoma ^***, ****^	10	0	0	0	0	0	0
No lesion	8	1	12.5	0	0	0	0

^*^Early cancer was defined as intramucosal cancer and submucosal invasive cancer

^**^Advanced adenoma: ≥ 10 mm and/or high-grade adenoma

^***^Non-advanced adenoma: < 10 mm and low-grade adenoma

^****^Advanced adenoma and non-advanced adenoma are aggregated with the highest grade of malignant lesion

Table 3 shows the values associated with the algorithm images produced and the quantitative fecal occult blood in group A. The sensitivity, specificity, accuracy, PPV, and NPV by sampling areas were 77.1%, 96.9%, 90.0%, 93.1%, and 88.7%, respectively, and by case were 85.0%, 96.7%, 92.0%, 94.4%, and 90.6%, respectively.

**Table 3:** The concordance between algorithm images and quantitative fecal occult blood values in Group A and Group B

	Fecal immunochemical test							
	Group A				Group B			
	Per sampling area (*n* = 100)		Per cases (*n* = 50)		Per sampling area (*n* = 250)		Per cases (*n* = 50)	
	≥ 400 ng/ml	< 400 ng/ml	≥ 400 ng/ml	< 400 ng/ml	≥ 400 ng/ml	< 400 ng/ml	≥ 400 ng/ml	< 400 ng/ml
Discrimination by algorithm								
Positive	27	2	17	1	45	14	13	4
Negative	8	63	3	29	9	182	2	31

### Algorithm accuracy verification

In the discriminant algorithm images for group B, a whole positive pattern, partial positive pattern, and whole negative pattern were found in 4, 13, and 33 cases, respectively (Fig. 2c, d, e).

Table 3 shows the values associated with the algorithm images produced and the quantitative fecal occult blood in group B. The sensitivity, specificity, accuracy, PPV, and NPV by sampling areas were 83.3% [95% confidence interval: 70.7–92.1], 92.9% [88.3–96.0], 90.8% [86.5–94.1], 76.3% [63.4–86.4], and 95.3% [91.2–97.8], respectively, and by case were 86.7% [59.5–98.3, 88.6% [73.3–96.8], 88.0% [75.7–95.5], 76.5% [50.1–93.2], and 93.9% [79.8–99.3], respectively.

Three of the four false positive cases were cancer cases. In other words, of the 17 positive cases in the discriminant algorithm images, 16 (94.1%) were cancer cases, accounting for 61.5% (16/26) of all cancer cases (Table 4). On the other hand, 14 (93.3%) of the 15 positive cases for fecal occult blood quantification of ≥ 400, 14 (93.3%) were cancer cases, accounting for 53.8% (14/26) of all cancer cases.

**Table 4:** The detected lesions by discriminant algorithm image and quantitative fecal occult blood values in Group B

	Cancer	Advanced adenoma *, ***	Non-advanced adenoma **, ***	No lesions
Discrimination by algorithm				
Positive	16	0	0	1
Negative	10	7	8	8
Quantitative fecal occult blood values				
≥ 400 ng/ml	14	0	0	1
< 400 ng/ml	12	7	8	8

* Advanced adenoma: ≥ 10 mm and/or high-grade adenoma

** Non-advanced adenoma: < 10 mm and low-grade adenoma

*** Advanced adenoma and non-advanced adenoma are aggregated with the highest grade of malignant lesion

### Verification of Hb-specific recognition

**Fig. 3:**
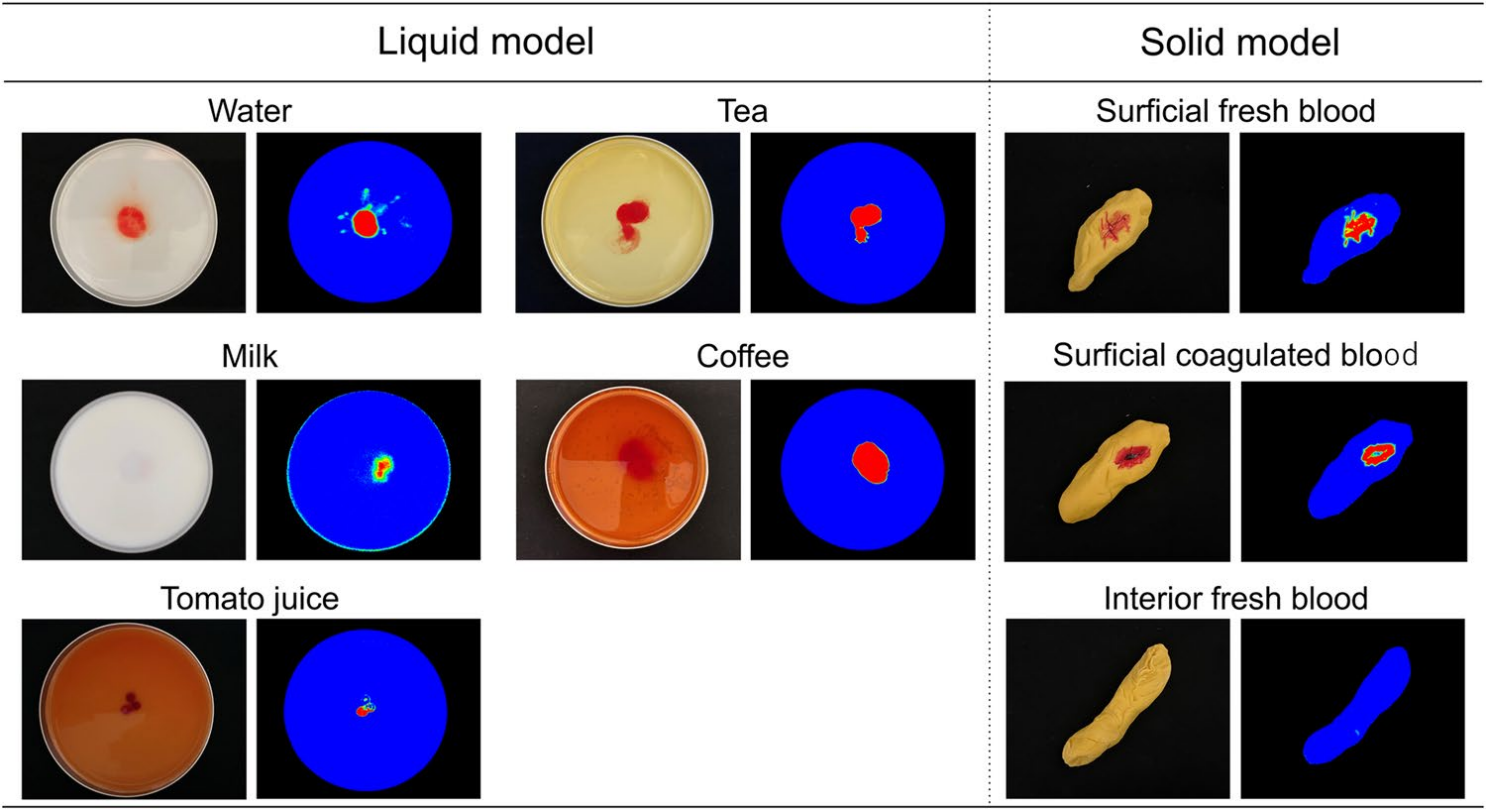
Verification image of Hb recognition accuracy

In various liquids (water, milk, tomato juice, tea, and coffee) in which small amounts of blood were dropped, only the blood was colored in the discriminant algorithm images (Fig. 3). Especially in milk, the blood was accurately imaged, although the blood precipitated and became unrecognizable from the surface. Stool models with blood on the surface were imaged with only blood, both fresh and coagulated blood; however, fresh blood inside the stool model were not imaged (Fig. 3).

## Discussion

This study was successful in imaging high quantitative values of fecal occult blood on the stool surface through HSI. To the best of our knowledge, this technology is the first of its kind in the world, and we are confident that this will have a significant impact on the development of medical devices in the future.

In the United States, the mortality rate of colorectal cancer has been declining since around 1980, largely attributed to screening tests such as fecal occult blood tests, sigmoidoscopy, and total colonoscopy, which are widely undergone by many United States citizens [18–20]. The effectiveness of colorectal cancer screening in preventing the disease is well recognized, and FIT-based colorectal cancer screening programs have been implemented in various countries [21]. FIT, a low-cost, noninvasive test, has been shown to correlate with lower colorectal cancer mortality rates despite having more false-negative results than colonoscopy [22, 23]. A meta-analysis in the United States and Europe demonstrated that fecal occult blood tests over a 2-day period have a sensitivity of 77% and specificity of 93% for detecting colorectal cancer [3]. In recent years, the development of new detection modalities for malignant diseases has been reported. These include liquid biopsy, which utilizes body fluid samples such as blood, urine, and stool [24, 25], and a novel method using N-NOSE [26]. However, none of these methods has provided data surpassing the utility of fecal occult blood testing in detecting colorectal cancer. Recently, it has been reported that a next-generation stool test kit, which detects trace amounts of colorectal cancer DNA, can identify 93% of colorectal cancers in a single fecal test [27]. Nevertheless, this test is similar to other tests that require the intention to undergo a checkup and is unlikely to reduce the number of people who remain unexamined.

However, in Japan, where the 2-day FIT method has been used for colorectal cancer screening since 1997, the mortality rate remains higher than those in Europe and the United States. In addition, the low screening rate has become a concern. Possible reasons for this may include (1) the lack of compulsion to take the test, (2) a population with low interest in health care, and (3) insufficient widespread understanding of the test's usefulness. Furthermore, the fecal occult blood test is time-consuming as it requires a sample to be scraped from the stool and necessitates innovative measures to increase the examination rate. Therefore, as the development of an imaging technology capable of rapidly and easily identifying occult blood in stools would address this issue, we explored detecting blood in stool using HSI. We hypothesized that if implemented, fecal occult blood could be instantly measured during routine bowel movements by integrating this technology into toilets.

Setting cutoff values is important in constructing an algorithm to discriminate blood in stool using HSI. The quantitative measurement of fecal occult blood for colorectal cancer screening has been extensively reported, and fecal occult blood quantitative values are particularly high when the cancer is at the advanced stage [10, 11]. In this study, we aimed to develop a toilet that would increase the number of people who receive screening and detect as many colorectal cancers as possible among those who do not receive screening. Therefore, instead of the commonly used screening cutoff value of 100 ng/ml [28–30], we set a higher cutoff value that would detect lesions at a high rate in daily bowel movements and have a low false positive rate. The sensitivity of colorectal cancer and adenoma lesions was compared, but no significant difference was found. Therefore, we utilized the 400 ng/ml cutoff value, assumed to yield fewer false negatives, for constructing the algorithm. The algorithm's precision was confirmed with impressive results: 90.0% discrimination accuracy for each analyzed region and 92.0% discrimination accuracy per case, indicating that HSI can discriminate high quantitative values of occult blood in stool, with high accuracy. Validation of variation further demonstrated high discrimination accuracy of 90.8% (95% confidence interval: 86.5–94.1%) by region and 88.0% (95% confidence interval: 75.7–95.5%) by case. High sensitivity and specificity were also observed. Furthermore, many of the false positive cases were also cancer cases, suggesting that if this discrimination image is positive, colon cancer would be detected at a high rate. In the future, we would like to continue our research and further improve analytical techniques so that I can image the blood at a cutoff value of 100 ng/ml, which is the level used in screening tests.

The advantage of HSI is that it can measure the entire stool. The images could be classified into three major categories: whole positive pattern, partial positive pattern, and whole negative pattern. Many of the results were partial positive patterns, suggesting that there may be an uneven distribution of fecal occult blood values even on the stool surface, which is conceivably a factor increasing the false-negative rate of conventional fecal occult blood tests where the participant collects the specimen.

In this study, blood was mixed with various liquids (water, milk, tomato juice, tea, and coffee) to verify whether HSI can specifically recognize Hb and not merely color, photographing only blood in all cases. Particularly with milk, even though the blood had settled and some parts of the blood serum were not visible to the naked eye, they could be depicted in the discriminant image. This indicates that Hb is identified in HSI not by color difference but as a characteristic of reflected and absorbed light wavelengths. Moreover, the ability to detect it even when passing through liquid suggests that detection in the toilet might also be feasible. However, in experiments using a stool model, while blood on the stool surface was detectable, blood within the stool remained unrecognized, suggesting to a potential limitation of the imaging technology. In the future, we plan to compare the detection ability of lesions in an RCT after creating a practical device to determine whether the inability to detect fecal occult blood in stool will be a limitation to the practical application of the device.

Though there have been numerous toilet studies conducted in recent years, such as those monitoring health by analyzing stool properties according to the Bristol Stool Scale [31] and those attempting to implement a system to indirectly measure blood glucose levels in urine using a color sensor [32], no studies have reported implementing technology to analyze blood in stool for colorectal cancer screening. This study suggests that detecting fecal occult blood in the stool during daily bowel movements could promptly inform patients about the presence of fecal occult blood on the stool surface without delay. Moreover, the ability to analyze the entire stool surface is expected to enhance accuracy compared to traditional fecal occult blood tests. Furthermore, the development of a toilet that alerts relevant parties in real-time to the need for further examination and encourages them to undergo screening or visit a medical institution has begun, aiming to facilitate early detection of colorectal cancer in individuals who have little interest in health care and do not participate in screening. If this toilet is developed, we plan to install it first in public facilities such as hotels and shopping centers. If many people with colorectal cancer are identified through the use of this toilet, it is expected that use of this toilet will increase, leading to an increase in the screening rate and ultimately a decrease in the colorectal cancer mortality rate.

The limitations of this study are as follows. First, since many of the study participants had cancer, only a few of them had no findings. Therefore, a large-scale prospective study is required for further validation of findings. Second, the imaging conditions used in the analysis involved photographs obtained under dark room conditions, not underwater. Additional studies are needed for clinical application to toilets, considering halation and light reflection during image processing when the feces are submerged in water.

This study demonstrates that HSI can facilitate the imaging of high quantitative values of fecal occult blood on stool surfaces, highlighting its potential for future clinical applications in toilets to aid in the prevention of colon cancer.
